# Endoscopic Laser Treatment for Pediatric Nasal Allergy

**DOI:** 10.1155/DTE.6.189

**Published:** 2000

**Authors:** Susumu Araki, Nobuhiro Suzuki, Harushiro Sato, Taro Yamaguchi, Hiroyuki Fujita, Yoko Umezawa, Mamoru Suzuki

**Affiliations:** Department of Otolaryngology Tokyo Medical University 6-7-1 Nishi-Shinjuku Shinjuku-ku Tokyo 160-0023 Japan

## Abstract

We have used the carbon dioxide (CO_2_) laser and the gallium aluminium arsenide (GaAlAs) diode laser with flexible fiber delivery instruments for vaporization of the inferior nasal turbinate in pediatric patients since 1993. Under endoscopic control, the whole inferior turbinate was vaporized by 5–10 W laser output delivered via an optical fiber. Generally, the nasal mucosa changes into normal mucosa, and symptoms improve. The greatest symptomatic improvement was in nasal obstruction. The results obtained by the two laser devices were similar although they have had different characteristics. Endoscopic laser surgery is effective in the treatment of pediatric nasal allergy.


*Diagnostic and Therapeutic Endoscopy*, Vol. 6, pp. 189–192
Reprints available directly from the publisher
Photocopying permitted by license only

© 2000 OPA (Overseas Publishers Association) N.V.
Published by license under
the Harwood Academic Publishers imprint,
part of The Gordon and Breach Publishing Group.
Printed in Malaysia.

### Endoscopic Laser Treatment for Pediatric Nasal Allergy

SUSUMU ARAKI*, NOBUHIRO SUZUKI, HARUSHIRO SATO, TARO YAMAGUCHI, HIROYUKI FUJITA, YOKO UMEZAWA and MAMORU SUZUKI

*Department of Otolaryngology, Tokyo Medical University, 6-7-1 Nishi-Shinjuku, Shinjuku-ku 160-0023, Tokyo, Japan*

*(Received 10 November 1999; In final form 17 January 2000)*

**We have used the carbon dioxide ($CO_2$) laser and the gallium aluminium arsenide (GaAlAs) diode laser with flexible fiber delivery instruments for vaporization of the inferior nasal turbinate in pediatric patients since 1993. Under endoscopic control, the whole inferior turbinate was vaporized by 5–10 W laser output delivered via an optical fiber. Generally, the nasal mucosa changes into normal mucosa, and symptoms improve. The greatest symptomatic improvement was in nasal obstruction. The results obtained by the two laser devices were similar although they have had different characteristics. Endoscopic laser surgery is effective in the treatment of pediatric nasal allergy.**

*Keywords:* $CO_2$ laser, Diode laser, Endoscospic laser surgery, Pediatric nasal allergy

### INTRODUCTION

Nasal allergy can be treated by surgical procedures such as inferior turbinectomy. However, it is difficult in children due to bleeding, discomforting nasal packing, difficulty in the selection of anesthesia, etc. On the other hand, laser devices have been effective for otolaryngologic surgery for allergic rhinitis in adults [1–6]. In pediatric cases, however, the laser technique needs to be modified with smaller instruments. We have used the carbon dioxide ($CO_2$) laser and the gallium aluminium arsenide (GaAlAs) diode laser with a flexible fiber delivery system for vaporization of the inferior nasal turbinate in pediatric patients. Table I indicates some of the common characteristics of two laser devices. The $CO_2$ laser is known for its long wavelength and shallow penetration depth. The diode laser has a short wavelength and deep penetration depth. Allergic reactions occur in the epithelial layer. By cauterizing only the surface of the nasal mucosa, the $CO_2$ laser would be suitable to treat nasal allergy. In hemorrhagic cases, however, the diode laser would be more suitable for treatment. Since previous studies have not compared $CO_2$

TABLE I Comparative study of surgical lasers

| | $CO_2$ | Diode |
|---|---|---|
| Wavelength(nm) | 10 600 | 805 |
| Absorbance by water | Large | Small |
| Absorbance by hemoglobin | Small | Small |
| Absorbance by blood | Large | Small |

* Corresponding author. Tel.: +81-3-3342-6111. Fax: +88-3-3346-9275. E-mail: susumu@tokyo-med.ac.jp.

and diode laser devices in terms of treatment for allergic rhinitis, this study attempts to evaluate them in terms of therapeutic effect.

### METHODS

The procedures were performed on 22 patients, 13 boys and 9 girls, ranging in age from 9 to 15 years (mean 12.4). Fifteen patients were treated with the $CO_2$ laser device, and 7 were with diode laser device. From 1993 to 1996, the $CO_2$ laser device was employed, and in 1997, the diode laser device was employed. All patients were given diagnoses of allergic rhinitis with multiple allergens based on results of radioallergosorbency tests. No drug treatment had been effective in any patient. Patients with other nasal diseases such as chronic sinusitis were excluded from the study.

In order to determine the optimum laser power, we studied the relationships between the laser power and the penetration depth in nasal mucosa after vaporization with both lasers at various power levels (1–10 W) for 0.5 s. Figure 1 indicates that the penetration depth of the $CO_2$ laser at from 2 to 9 W was approximately 0.6 mm. Figure 1 also indicates a similar depth of penetration with the diode laser with $CO_2$ laser (0.6 mm) producing from 7 W up. Because of these results, we chose 6–10 W power for diode laser treatment.

The patients underwent preoperative infiltrative anesthesia using small pieces of gauze impregnated with 4% lidocaine and 0.02% epinephrine.

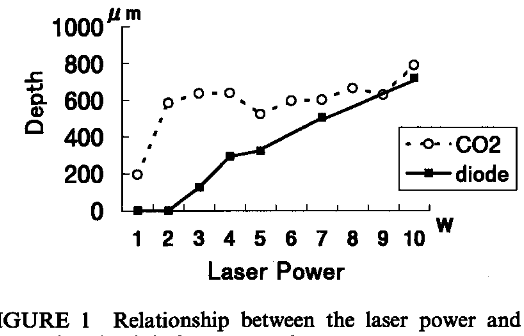

FIGURE 1 Relationship between the laser power and the penetration depth in human nasal mucosa.

Neither general anesthesia nor hospitalization was required.

Using the $CO_2$ laser device (Mochida Pharmacentical Co. Ltd., Tokyo, Japan), the surgeon can have a flexible optical fiber with a diameter of 2.3 mm (Fig. 2). For non-contact mode procedures, a 5–7 W, continuous mode, defocused laser beam was delivered. Using the diode laser device (Diomed, Cambridge, UK), the surgeon can use a flexible fiber with a diameter of 0.4 mm (Fig. 3). For contact mode procedures, a 6–10 W, continuous mode, defocused laser beam was employed.

All laser procedures were performed under endoscopic monitoring via a video system. The diameters of the endoscopes were 4 and 2.7 mm. This system including the built-in filter for the diode laser was

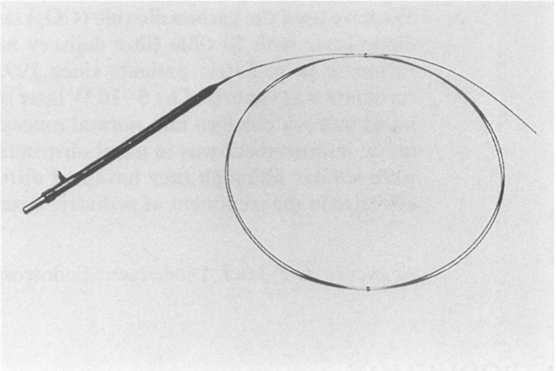

FIGURE 2 The disposable flexible optical fiber for the defocused $CO_2$ laser beam.

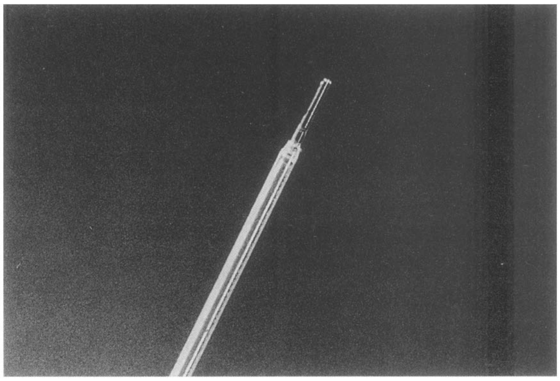

FIGURE 3 A flexible quartz fiber for the defocused diode laser beam (0.5–60 W).

TABLE II Patients questionnaire

| | |
|---|---|
| How do you usually breathe? (nasal obstruction) | 1. Always through the nose<br>2. No mouth breathing<br>3. Sometimes through the mouth<br>4. Always through the mouth |
| How many times a day do you blow your nose? (nasal discharge) | 1. None<br>2. Less than 5<br>3. 5–9<br>4. More than 9 |
| How many times a day do you sneeze? | 1. None<br>2. Less than 5<br>3. 5–9<br>4. More than 9 |

assembled by Olympus Optical Co. (Tokyo, Japan). Eye protection for the observers was not necessary with the monitoring video system.

To evaluate overall improvement, all patients filled in a questionnaire before and more than 2 weeks after the procedure (Table II). In the evaluation of nasal obstruction, grades 4 and 3 mean that the patient had marked symptoms. Grade 2 indicates slight symptoms, and grade 1 indicates no symptoms. Rhinorrhea was evaluated by the number of times they blew their noses per day. The evaluation of sneezing was the same as that for rhinorrhea. The symptomatic improvements were classified as excellent (preoperative score minus post-treatment score grades = 2), good (1), unchanged, and worse. Percent improvement was calculated to compare $CO_2$ and diode laser treatments: Percent improvement = 100 × [(excellent response cases + good response cases)/total number]. Statistical differences of percent improvements between $CO_2$ and diode laser groups were evaluated by $\chi^2$ test.

### RESULTS

Typical changes after laser treatment are shown in Fig. 4. A 13-year-old girl with allergic rhinitis had a chief complaint of nasal obstruction. Preoperatively the mucosa of the inferior turbinate was pale and edematous. Under endoscopic control, the whole inferior turbinate was cauterized by the $CO_2$

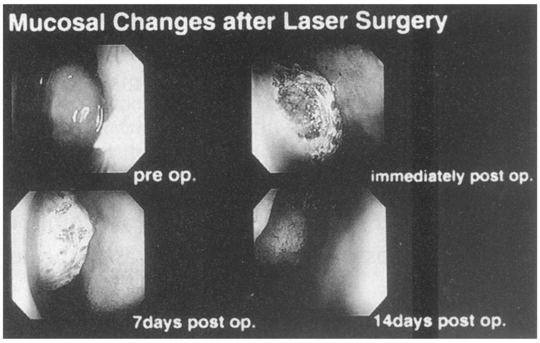

FIGURE 4 Seven watt continuous mode $CO_2$ laser beam was used for the surgery.

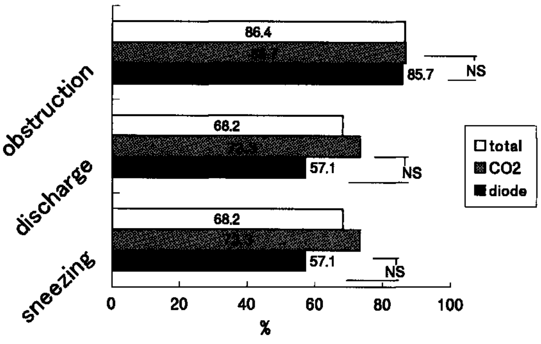

FIGURE 5 Comparison of percent improvement after surgery between the $CO_2$ and the diode laser in 22 patients. NS = no significant difference.

laser using a power output of 7 W, anteriorly then posteriorly and then inferiorly. By 2 weeks after the laser treatment, the mucosal color had become normal, and the edema of the inferior turbinate improved.

Figure 5 indicates percent improvements of three symptoms after laser treatment in 22 patients. Concerning symptoms of nasal obstruction, 12 out of 22 showed excellent results (54.5%), 7 showed good results (31.8%), 3 were unchanged (13.6%), and none showed a worse result (0%). Concerning improvement of sneezing, 8 out of 22 showed excellent results (36.4%), 7 showed good results (31.8%), 6 were unchanged (27.3%), and 1 showed a worse result (4.5%). Concerning improvement of nasal discharge, 4 out of 22 showed excellent results (18.2%), 11 showed good results (50.0%), 7 were unchanged (31.8%), and none worsened (0%). Percent improvement of nasal obstruction (86.4%) was higher than the other two symptoms. There was no significant difference between the $CO_2$ and the diode laser groups (nasal obstruction: $\chi^2 = 0$, $P = 0.999$; nasal discharge: $\chi^2 = 0.072$, $P = 0.789$; sneezing: $\chi^2 = 0.072$, $P = 0.789$). There were no complications, such as postoperative bleeding, after the laser procedure.

### DISCUSSION

Lippert and Werner [6] compared the results of inferior turbinectomy in children and adults between the $CO_2$ and neodymium : yttrium–aluminum-garnet lasers (Nd : YAG). Their study indicated that both lasers were useful tools for the reduction of hyperplastic inferior nasal turbinates. The present study in children showed similar results in the treatment of allergic rhinitis. Lippert and Werner [6] reported that postoperative care after Nd : YAG laser therapy takes longer than that following $CO_2$ laser treatment. Because of more similar characteristics of the diode laser and the Nd : YAG laser, we thought that postoperative care after diode laser procedure might be difficult in children. However, the present result showed no significant difference between $CO_2$ and diode laser treatment. Figure 1 indicated that similar depths of vaporization (0.6 mm) were produced with the diode laser and with the $CO_2$ laser at power output levels from 7 to 10 W. This is related to the similar clinical results obtained.

One of the differences between the previously reported Nd : YAG laser treatment and our diode laser treatment was the laser power. We chose a lower power of 10 W or less. On the other hand, Lippert and Werner [6] employed higher power (5–30 W). High power results in extensive and deep vaporization, and may damage the periosteum. If the diode laser were applied at a power of over 10 W, the tissue may be vaporized deeply, and postoperative bleeding or crust formation may continue for a long time.

There was no case of postoperative complication in our treatment. Low laser power and accurate vaporization produce less bleeding, and prevent adhesion of nasal mucosa. Endoscopy is therefore necessary for the procedure. In conclusion, endoscopic laser treatment for pediatric allergic rhinitis is effective and safe.

### *Acknowledgments*

The authors wish to thank Dr. Kouji Otsuka, Dr. Kouichi Kitamura, Dr. Hideaki Takagi, Dr. Tatsuya Hasegawa and Dr. Fumika Hamada for cooperation in performing laser treatment. The authors are also grateful to Professor J. Patrick Barron of the International Medical Communication Center of Tokyo Medical University for reviewing the manuscript.

### *References*

[1] Mittelman, H. $CO_2$-laser turbinectomies for chronic, obstructive rhinitis. *Lasers Surg. Med.* 1982; **2**: 29–36.

[2] McCombe, A.W. and Jones, A.S. A comparison of laser cautery and sub-mucosal diathermy for rhinitis. *Clin. Otolaryngol.* 1992; **17**: 297–299.

[3] Kawamura, S., Fukutake, T., Kubo, N. *et al.* Subjective results of laser surgery for allergic rhinitis. *Acta Otolaryngol. Suppl.* (*Stockh.*) 1993; **500**(Suppl.): 109–112.

[4] Dilkes, M.G., Cameron, I., Quinn, S.J. *et al.* Preliminary experience with an 810 nm wavelength diode laser in ENT surgery. *Laser Med. Sci.* 1994; **9**: 261–264.

[5] Ito, H., Baba, S., Suzuki, M. *et al.* Severe perennial allergic rhinitis treated with Nd : YAG laser. *Acta Otolaryngol.* (*Stockh.*) 1996; **525**(Suppl.): 14–17.

[6] Lippert, B.M. and Werner, J.A. Comparison of carbon dioxide and neodymium : yttrium–aluminum-garnet laser in surgery of the inferior turbinate. *Ann. Otol. Laryngol.* 1997; **106**: 1036–1042.

